# DNA methylation alterations across time and space in paediatric brain tumours

**DOI:** 10.1186/s40478-022-01406-8

**Published:** 2022-07-16

**Authors:** Anna Wenger, Sandra Ferreyra Vega, Elizabeth Schepke, Maja Löfgren, Thomas Olsson Bontell, Magnus Tisell, Daniel Nilsson, Teresia Kling, Helena Carén

**Affiliations:** 1 Sahlgrenska Center for Cancer Research, Department of Laboratory Medicine, Institute of Biomedicine, Sahlgrenska Academy, University of Gothenburg, Medicinaregatan 1F, 405 30 Gothenburg, Sweden; 2 Department of Clinical Neuroscience, Institute of Neuroscience and Physiology, Sahlgrenska Academy, University of Gothenburg, Gothenburg, Sweden; 3 Childhood Cancer Centre, Queen Silvia Children’s Hospital, Sahlgrenska University Hospital, Gothenburg, Sweden; 4 Department of Physiology, Institute of Neuroscience and Physiology, Sahlgrenska Academy, University of Gothenburg, Gothenburg, Sweden; 5 Department of Clinical Pathology, Sahlgrenska University Hospital, Gothenburg, Sweden; 6 Department of Neurosurgery, Sahlgrenska University Hospital, Gothenburg, Sweden

**Keywords:** Childhood cancer, DNA methylation, Brain tumour, EPIC methylation array, Heterogeneity, Intratumour, Classification, Relapse

## Abstract

DNA methylation is increasingly used for tumour classification and has expanded upon the > 100 currently known brain tumour entities. A correct diagnosis is the basis for suitable treatment for patients with brain tumours, which is the leading cause of cancer-related death in children. DNA methylation profiling is required for diagnosis of certain tumours, and used clinically for paediatric brain tumours in several countries. We therefore evaluated if the methylation-based classification is robust in different locations of the same tumour, and determined how the methylation pattern changed over time to relapse. We sampled 3–7 spatially separated biopsies per patient, and collected samples from paired primary and relapse brain tumours from children. Altogether, 121 samples from 46 paediatric patients with brain tumours were profiled with EPIC methylation arrays. The methylation-based classification was mainly homogeneous for all included tumour types that were successfully classified, which is promising for clinical diagnostics. There were indications of multiple subclasses within tumours and switches in the relapse setting, but not confirmed as the classification scores were below the threshold. Site-specific methylation alterations did occur within the tumours and varied significantly between tumour types for the temporal samples, and as a trend in spatial samples. More alterations were present in high-grade tumours compared to low-grade, and significantly more alterations with longer relapse times. The alterations in the spatial and temporal samples were significantly depleted in CpG islands, exons and transcription start sites, while enriched in OpenSea and regions not affiliated with a gene, suggesting a random location of the alterations in less conserved regions. In conclusion, more DNA methylation changes accumulated over time and more alterations occurred in high-grade tumours. The alterations mainly occurred in regions without gene affiliation, and did not affect the methylation-based classification, which largely remained homogeneous in paediatric brain tumours.

**Supplementary Information:**

The online version contains supplementary material available at 10.1186/s40478-022-01406-8.

## Introduction

Brain tumours are the most common cause of cancer-related death in children and the second most common type of paediatric cancer [27, 38]. More than 100 types of central nervous system tumours (brain and spinal cord) exist and the prognosis and treatment vary largely between the diagnoses [22, 27]. Pilocytic astrocytoma, the most common type of low-grade glioma (LGG), has, for instance, a 5-year relative survival of 97% [27]. In contrast, survival for medulloblastoma (a high-grade embryonal tumour) varies largely between and within [5, 27] (< 50–90% 5-year survival) [33] the principal four molecular subgroups recognized in the World Health Organisation (WHO) 2021 classification; WNT (wingless)-activated, SHH (sonic hedgehog)-activated and *TP53*-mutated, SHH-activated and *TP53*-wildtype and non-WNT/non-SHH (consisting of the so-called Group 3 and Group 4 classes) [13, 22, 39]. These subgroups are determined by DNA methylation analysis, which has been shown increasingly important for tumour classification, subtyping and biomarkers such as *0‐6‐Methylguanine DNA‐methyltransferase* (*MGMT*) [3, 11, 16]. Moreover, methylation-based classification has been introduced in the clinic for all paediatric brain tumours in many countries and more will likely follow [3, 4, 30, 31] (accepted manuscript Schepke et al.).

We recently showed that the methylation subclass, according to the largest existing methylation-based classifier [3], differs within single adult-type glioblastoma (GBM) tumours, and that the *MGMT* promoter methylation status, a prognostic and therapy predictive biomarker, also varied intratumourally [44]. It is therefore essential to verify the robustness of the methylation-based classification to ensure homogeneous diagnosis of the paediatric tumours. We aimed to profile the spatial intratumour methylation pattern in paediatric brain tumours, and its implications for methylation-based classification. We also studied the temporal heterogeneity in paired primary and relapse tumours to generate knowledge on methylation alterations at recurrence. Our analysis of 49 spatial and 72 temporal paediatric brain tumour samples overall showed a largely stable methylation pattern and methylation-based classification across time and space.

## Materials and methods

### Patients and samples

Signed informed consent was obtained from the guardians of paediatric patients undergoing brain tumour resection 2018–2021 at Sahlgrenska University Hospital (Gothenburg, Sweden; Dnr: 604-12, Regional Ethical Review Board in Gothenburg). Three to seven spatially separated biopsies per patient (n = 11) were sampled, imprints were made and the tissue was fresh-frozen in liquid nitrogen for subsequent DNA extraction. Samples were also collected from a single location of 35 paired, primary and relapse, paediatric tumours as described above or from the Swedish National Methylation Study (accepted manuscript Schepke et al.; Dnr: 604.12, T1162-16; sample collection 2017–2020 and inclusion of paired primary tumour tissue from several years prior). Note that the relapse samples for two patients (pTT-3 and pTT-18) are from a metastasis, and pTT-35 are both relapse samples. pTT-26 is a paired biopsy and operation (almost 10 months apart), but was included anyway as a temporal sample. The tumour tissue from the patients in the national study was formalin-fixed and paraffin-embedded (FFPE) prior to DNA extraction.

### DNA methylation analysis

DNA was extracted from fresh-frozen tumours as previously described [18]. DNA extraction from FFPE samples was performed with the Maxwell® RSC system using the FFPE Plus DNA Kit reagents and protocol (Promega, Madison, WI, USA). Bisulfite-modified DNA (Zymo, Orange, CA, USA) from FFPE samples was restored with the Infinium HD FFPE DNA Restore Kit (Illumina, San Diego, USA), and then processed on the Infinium Methylation EPIC BeadChip together with the bisulfite-modified fresh-frozen samples (Illumina) according to the manufacturer’s instructions. Analysis of the resulting methylation data was performed with the statistical software R [34] as previously described [44] using ChAMP [23] and minfi [2] for processing the raw methylation data into β-values and normalisation with noob [9] and BMIQ [40]. Data from a public dataset containing 450K methylation array data of paired medulloblastoma and metastasis samples was also used; GSE63669 [43].

Classification of tumours was performed with the Molecular Neuropathology (MNP) classifier (https://www.molecularneuropathology.org/mnp/) [3], with its newest (unpublished) version 12.5. The classifier classifies samples into a methylation superfamily (e.g. medulloblastoma), and then further into a family (e.g. medulloblastoma non-WNT/non-SHH activated), a class (e.g. medulloblastoma group 4) and a subclass (e.g. medulloblastoma, non-WNT/non-SHH, Group 4, subclass V; referred to here as subclassification). At all levels, a calibrated classification score ≥ 0.9 (score ranging between 0 and 1) was considered a successful classification according to the instructions of the classifier. Samples with a calibrated score < 0.3 at the superfamily level were denoted here as “No classification” and presented with the classification that received the highest calibrated score. Previous classifier versions (v11b2 and 11b4) had a cut-off at ≥ 0.5 for successful subclassification. V11b2 and 11b4 were used to analyse intratumour heterogeneity regarding methylation subclass in adult GBM [44], and v12.5 in LGG studies [8], which are referenced in this study. Copy-number alterations (CNAs) were inferred from the methylation data using conumee [15]. Heterogeneity in CNA between samples from the same patient (spatial and temporal patients) was determined by visual inspection. Differentially methylated positions (DMP; Δβ > 0.3) were calculated as previously described [44]. Prior to DMP analysis, we removed CpG sites in regions with homozygous deletions (CNA segment < − 0.4 as suggested previously [4, 37]) with a CNA-filter. The R-package survival was used to test if the time until relapse was predictive of the number of temporal DMPs with a Cox proportional hazards regression test.

Phylogenetic analysis was performed by calculating the Euclidean distance of the methylation data of the top 5000 variable probes for each patient. A normal paediatric brain sample (from GSE52556 [17]) was included in the distance calculation, but not in the selection of probes. The phylogenetic trees were inferred from the distances using a minimal evolution method [7] in the R-package ape [29].

### Histology and tumour cell content analysis

FFPE sections (temporal samples) or tumour imprints of fresh tumour tissue (spatial samples) were stained with hematoxylin and eosin and evaluated for tumour cell content by a specialist in clinical neuropathology. We also estimated the tumour cell content based on methylation data using the R-package InfiniumPurify [32]. The package can be used for LGG and GBM tumours respectively, but additional tumour types are not included in the InfiniumPurify estimator. For the remaining tumour types (ependymoma, medulloblastoma etc.), we therefore used histology to evaluate the tumour cell content.

## Results

### Methylation superfamily classification in paediatric brain tumours is stable

**Fig. 1:**
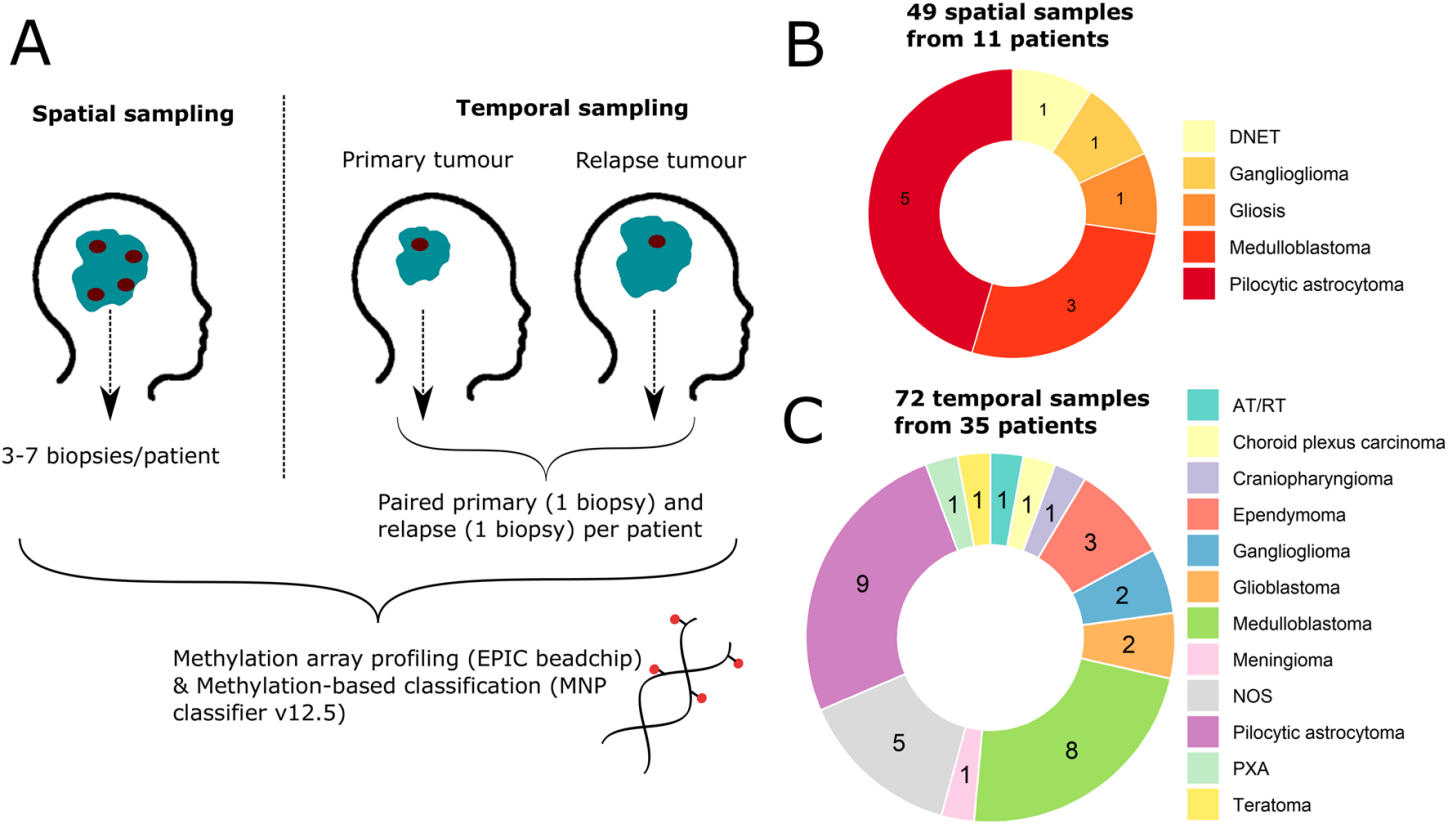
Experimental setup and patient cohort. **A** Children operated for a brain tumour were included in this study. We either sampled 3–7 spatially separated biopsies from the same tumour (left), or one biopsy from a primary tumour and one biopsy from the paired relapse tumour (right). All samples were processed on Infinium EPIC BeadChip methylation arrays and classified with the MNP classifier v12.5. **B** The pathological-anatomical diagnosis for the 11 patients in the spatial study setting, and **C** primary tumour of the 35 patients in the paired primary-relapse setting. *AT/RT* Atypical teratoid rhabdoid tumour, *NOS* Not otherwise specified, *PXA* Pleomorphic xanthoastrocytoma

**Fig. 2:**
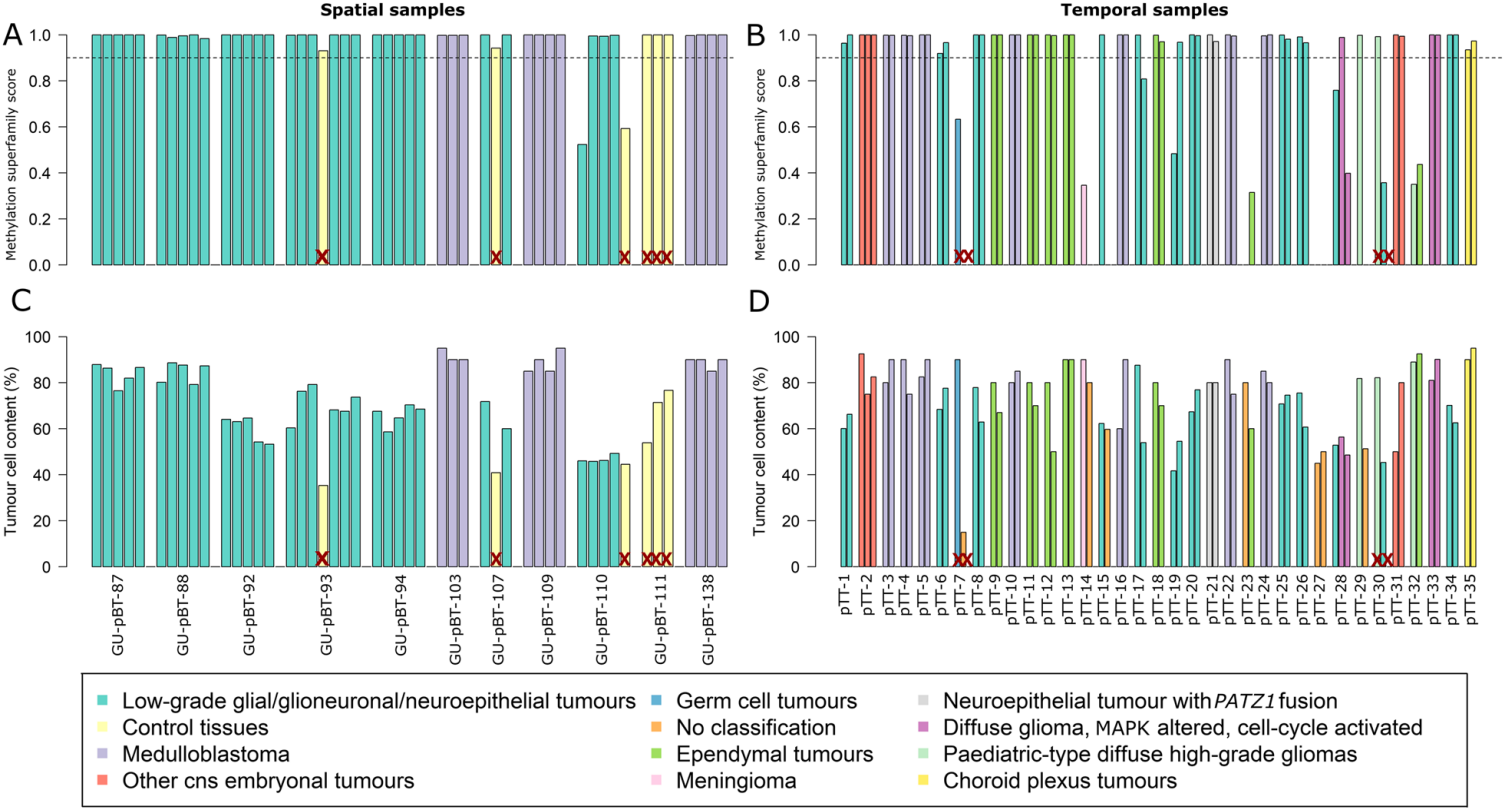
Methylation-based superfamily classification is robust for spatial and temporal paediatric brain tumours. **A** All samples were classified with the MNP classifier v12.5 and the calibrated classification score (≥ 0.9 is a successful classification; indicated by dashed line) is visualised on the y-axis for each spatial and **B** temporal sample. The samples are grouped according to their patient identity (x-axis) and coloured according to their best-predicted methylation superfamily. **C** The tumour cell content (y-axis) for spatial and **D** temporal samples. A red X denotes that the sample was excluded from further analysis. The legend applies to all subfigures

We sampled 3–7 spatially separated biopsies from 11 paediatric brain tumours, and paired primary and relapse samples from 35 patients, and processed all 121 samples on Infinium EPIC BeadChip methylation arrays (Fig. 1). No sample mixups were identified from analysis of single nucleotide polymorphisms included on the arrays thus verifying the patient identity of the samples (Additional File 1). All samples were next classified with the MNP methylation-based classifier [3] v12.5 into a methylation superfamily with a classification score, ranging from 0–1. One hundred two of the 121 samples (84%) were successfully classified (calibrated score ≥ 0.9; Fig. 2A, B), and the majority of the methylation superfamilies (medulloblastomas, ependymomas etc.) were consistently classified well (calibrated score > 0.9) whereas high-grade gliomas (HGG), LGG and diffuse gliomas had a larger spread in calibrated score (Additional File 2A). Samples with a lower tumour cell content (as low as ~ 45%) could still be matched to a methylation superfamily (Fig. 2C, D, Additional File 2B). As expected, the methylation superfamilies largely clustered together (Additional File 2C, D. Before further analysis, we excluded samples classified as control tissue. Samples classified with a score below 0.9 were inspected for tumour cell content and lack of CNAs for normal tissue content, and compared to histopathological diagnosis to examine if a reasonable methylation superfamily was called, in which case the samples were included for further analysis. The paired primary/relapse tumour to an excluded sample was also removed. In total, 111 of the 121 samples were included for detailed methylation analysis. Their methylation superfamily classification was in general in good agreement with the histopathological diagnosis, and in some cases able to provide a more specific diagnosis (Additional File 3). Exceptions did occur; e.g. pTT-13, with pathological-anatomical diagnosis (PAD) as an embryonal tumour was classified by methylation as an ependymoma *ZFTA* fusion-positive tumour with very high confidence (calibrated score 0.99).

### Site-specific methylation differences occur in the spatial setting at random locations

**Fig. 3:**
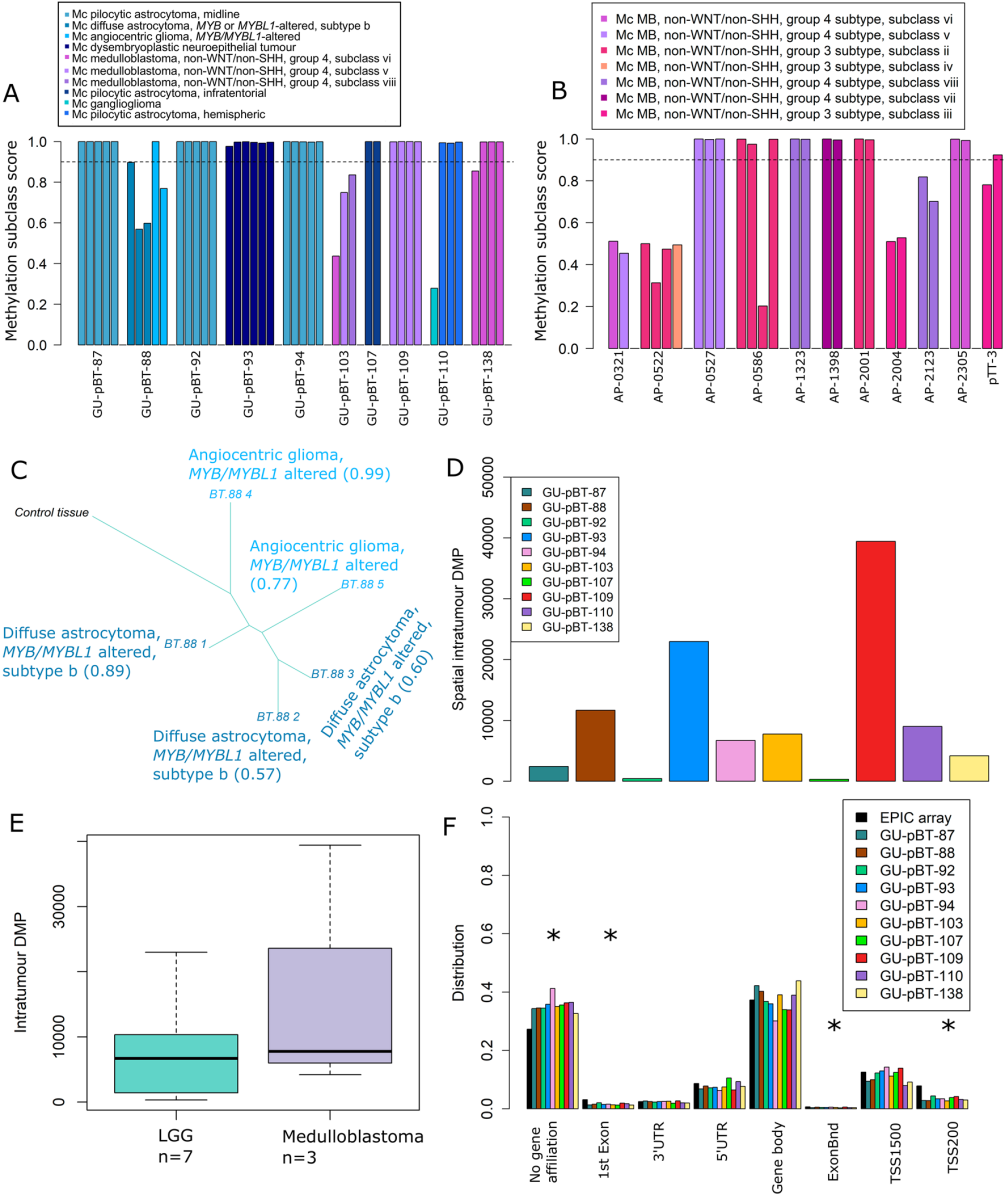
Methylation subclasses are homogeneous spatially, but site-specific alterations occur. **A** The methylation subclasses (≥ 0.9 is successfully classified; indicated by dashed line) of the included spatial samples are homogeneous for each patient, when considering the 0.9 threshold. **B** The subclasses were also stable in a cohort of paired medulloblastoma metastases. The first bar indicates the primary tumour and subsequent bars represent metastases. **C** Phylogenetic tree based on the top 5000 most variable CpG sites for GU-pBT-88 with a paediatric brain control tissue as reference. The phylogenetic trees demonstrate the evolutionary relationship between samples, and which samples that are the most different from each other. The colour denotes the methylation subclass and its calibrated score is written out in parenthesis. GU-pBT-88 has an indication of multiple subclasses, but not confirmed as the calibrated scores are below 0.9. **D** Differentially methylated positions (DMPs) were identified within each tumour by pairwise comparison of the intratumour biopsies, where a difference in β-value > 0.3 was considered a DMP. The number of DMPs varied between the tumours, and **E** there was a trend of more DMPs in medulloblastomas than low-grade gliomas. **F** The DMPs were significantly enriched in regions that were not associated with a gene and decreased in 1st Exon, Exon boundaries and TSS200 (transcription start site and 200 bp away) regions compared to the distribution of the array (first black bar). * denotes significant *p*-value < 0.01

We next focused on the spatial samples to determine how the methylation pattern differs within the tumours. First, we noted that the samples from medulloblastomas and LGG respectively clustered together (Additional File 4A). Also, the samples from each patient clustered together showing that the methylation profiles are more similar to each other than to tumour samples from other patients. We then inspected the methylation-based subclassification of the samples and it was homogeneous for the spatial samples in our cohort as well as a public dataset of paired primary medulloblastoma tumours and metastases (Fig. 3A, B). Three of the spatially sampled tumours (GU-pBT-88, GU-pBT-103 and GU-pBT-110) had an indication of multiple methylation subclasses, but not confirmed as the classification scores were below 0.9 (Fig. 3C, Additional File 4B, C).

Next, we focused on methylation differences spatially within the tumours and calculated the number of DMPs in the spatial study cohort. A site was considered a DMP if the β-value differed more than 30% between two samples of the same tumour. CpG sites located in regions with homozygous deletion were removed prior to the analysis (Additional File 4D). The number of intratumour DMPs varied substantially between the patients (min: 300, max: 39,400) and the medulloblastoma had on average more DMPs than the LGG (17,100 vs 7600), but the difference was not significant (Fig. 3D, E). The number of DMPs for LGG and medulloblastoma corresponded to 1.0–2.3% of the CpG sites on the array retained for analysis (728,898 CpG sites). There was no significant correlation between the intratumour difference in tumour cell content and the number of DMPs (r =  − 0.07, *p*-value = 0.85).

The DMPs mainly occurred within one biopsy pair within the tumours (rather than a shared DMP between several biopsies), and very few DMPs were shared between patients (Additional File 4E). The location of DMPs were significantly enriched in regions without gene affiliations and in so-called OpenSea regions (> 4 kb from a CpG island), and significantly decreased in 1st Exon regions, TSS200 (transcription start site + 200 bp) and CpG islands (Fig. 3F, Additional File 4F). Taken together, this suggests that DMPs in the spatial setting do occur within paediatric brain tumours, and that they are randomly located in less conserved regions.

We next inspected if the CNA profiles differed spatially within the tumours and found heterogeneity in 1 out of 7 LGG (GU-pBT-93). The LGG samples overall had very flat CNA profiles, except for GU-pBT-93_7, which had gain of chromosome 12 (Additional File 5A). As expected, the medulloblastoma samples had more CNAs than the LGGs and two of the patients had gains of *MYCN* and loss of *TP53*, and both markers were homogeneous in all biopsies. However, 2 out of 3 medulloblastoma (GU-pBT-109 and GU-pBT-138) differed in overall CNA profile e.g. with gain of chromosome arm 1q and focal deletion of 8p (Additional File 5B).

### Methylation classification is stable over time

**Fig. 4:**
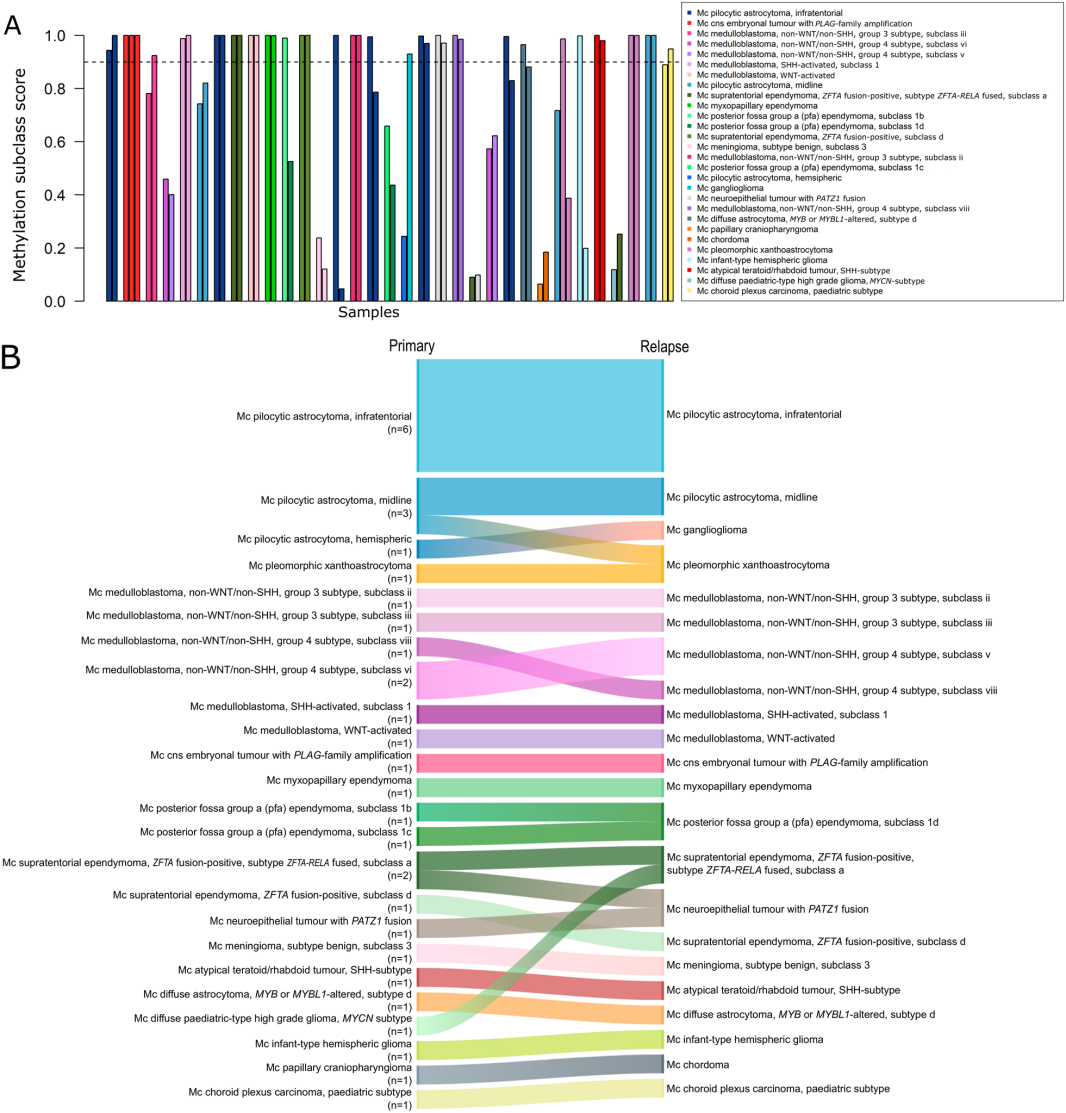
Methylation subclassification is stable over time. **A** The methylation subclass calibrated scores (y-axis) for all included temporal samples, where ≥ 0.9 is a successful classification (indicated by dashed line). There are no subclass switches for successfully classified samples, but indications of if samples with calibrated score < 0.9 are considered. The samples are grouped according to their patient identity (primary tumour to the left, recurrent tumour to the right) and the colour indicates the methylation subclass that received the highest calibrated score (top subclass) for each sample even if that score was below the 0.9 classification threshold. Two patients have three samples each; pTT-2 where the third sample is a metastasis, and pTT-28 which had a second relapse. **B** Sankey plot over the top subclass for the primary tumour (left) and relapse tumour (right). Note that samples are included in this plot even if the calibrated score is below the 0.9 threshold

The subclassification of the paired primary and relapse samples was homogeneous for each patient with no subclass switches with calibrated scores ≥ 0.9 (Fig. 4A, B). There were however indications (calibrated score < 0.9) of potential subclass changes for a few tumours; mainly within the medulloblastoma Group 4 subtype and the ependymoma posterior fossa group A family. There was also a discrepancy for one patient (pTT-28) between the PAD (pilocytic astrocytoma) and the methylation-based classification of the relapse tumours (pleomorphic xanthoastrocytoma; PXA). We next inspected the patients which did not have the exact same PAD or methylation class in the relapse compared to the primary tumour (Table 1). The methylation class was used here instead of the subclass as it is more similar to the WHO classification and e.g. the medulloblastoma subtype I–VIII switches (indicated on the subclass level) are not used clinically. Overall, the methylation classification was very stable, even when there were changes in the histopathological assessment, and remained the same in the relapse as in the primary tumour (Table 1).

**Table 1:** Changes/refinements in PAD or methylation class between the primary and relapse tumour

Patient ID	Primary PAD	Relapse PAD	Primary methylation class	Relapse methylation class
pTT-6	PA grade I	DLGNT	Supratentorial midline PA (0.74)	Supratentorial midline PA (0.82)
pTT-9	Malignant glioneural tumour	Anaplastic EPN grade III	Supratentorial EPN, *ZFTA:RELA* fusion-positive (0.99)	Supratentorial EPN, *ZFTA:RELA* fusion-positive (0.99)
pTT-19	Ganglioglioma	Ganglioglioma	Supratentorial PA (0.24)	Ganglioglioma (0.92)
pTT-21	PA grade I	Malignant high-grade tumour	Neuroepithelial tumour with *PATZ1* fusion (0.99)	Neuroepithelial tumour with *PATZ1* fusion (0.97)
pTT-23	High-grade primitive tumour	Malignant-high grade tumour	No class	No class
pTT-28	PA grade I	PA grade I	Supratentorial midline PA (0.71)	PXA(-like) (0.98)
pTT-32	Glioblastoma	Malignant high-grade tumour	Diffuse paediatric-type high grade glioma, MYCN subtype (0.11)	Supratentorial EPN, *ZFTA:RELA* fusion-positive (0.26)

The number in parenthesis states the calibrated score of the methylation classification. *PAD* Pathological anatomical diagnosis, *PA* Pilocytic astrocytoma, *DLGNT* Diffuse leptomeningeal glioneuronal tumour, *EPN* Ependymoma, *PXA* Pleomorphic xanthoastrocytoma

### Methylation-based classification of medulloblastoma family and class is homogeneous across time and space

**Fig. 5:**
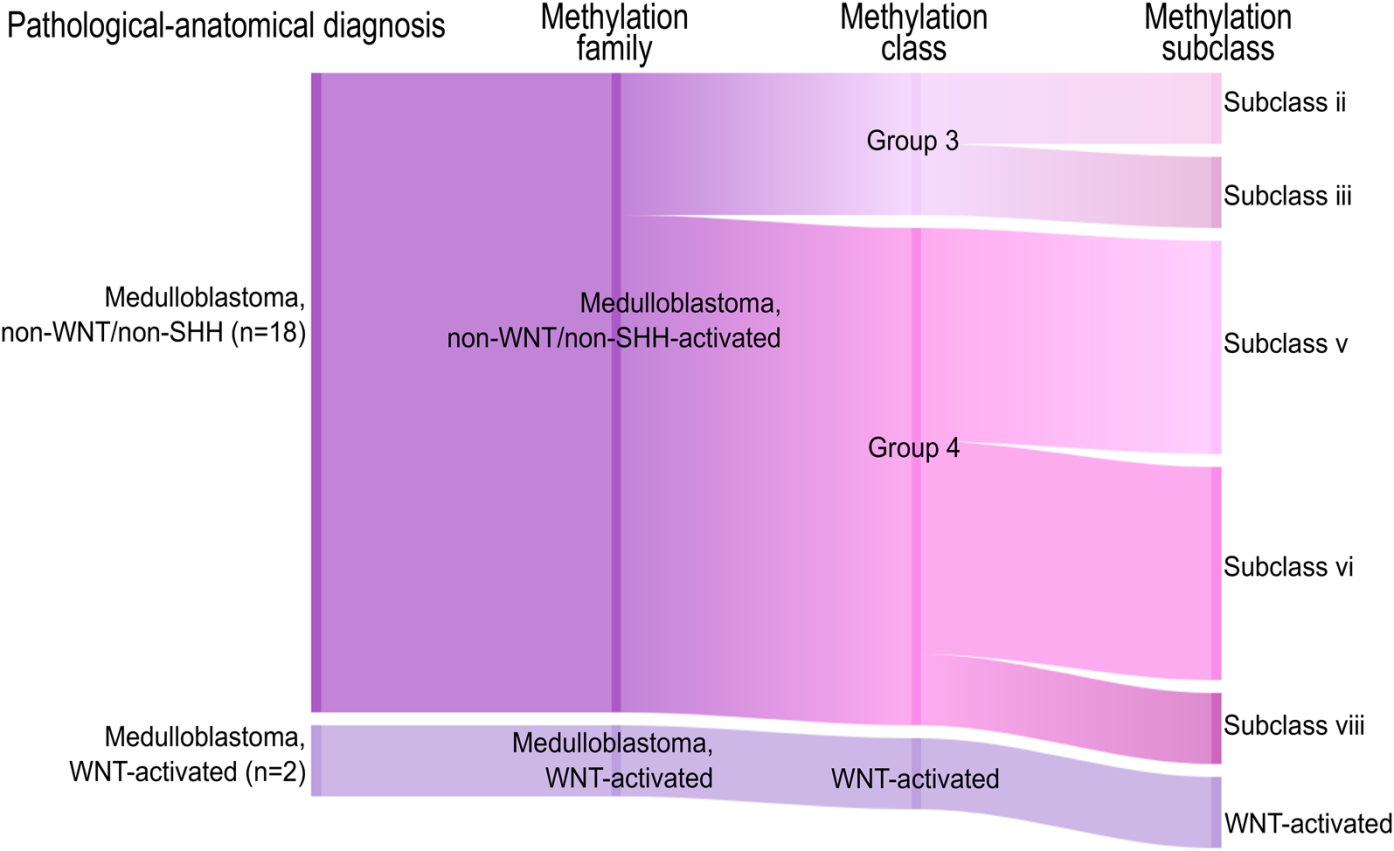
Methylation-based classification of medulloblastoma is stable on all classification levels across time and space. Sankey plot of the medulloblastoma subgroup, determined without DNA methylation, based on the pathological-anatomical diagnosis (left), followed by the top methylation family, methylation class and subclass (right). The plot includes all spatial and temporal (primary and relapse) medulloblastoma samples where the subgroup had been determined in the clinic prior to DNA methylation classification

Given the importance of methylation-based profiling for subgrouping of medulloblastoma, we focused in particular on the classification of these tumours in comparison to the subgrouping that had been performed according to clinical routine (e.g. histopathological stainings, immunohistochemistry, FISH and next-generation sequencing-based panels) for the PAD. We included both spatial and temporal (primary and relapse) medulloblastoma samples in this analysis and studied the methylation family (e.g. non-WNT/non-SHH), class (e.g. Group 4) and subclass (e.g. subclass VIII) level (Fig. 5). In all cases, the clinical subgrouping was in agreement with the methylation family and class. As expected, several subclasses (subclass I-VIII) were detected among the non-WNT/non-SHH samples, but these subclasses are currently not in clinical use. We also investigated the samples from each medulloblastoma patient (spatial and temporal), and found no heterogeneity on any of the methylation classification levels with calibrated scores > 0.9. Methylation classification of medulloblastoma was thus stable across time and space in our cohort (n = 10 patients).

### Site-specific methylation alterations accumulate over time

**Fig. 6:**
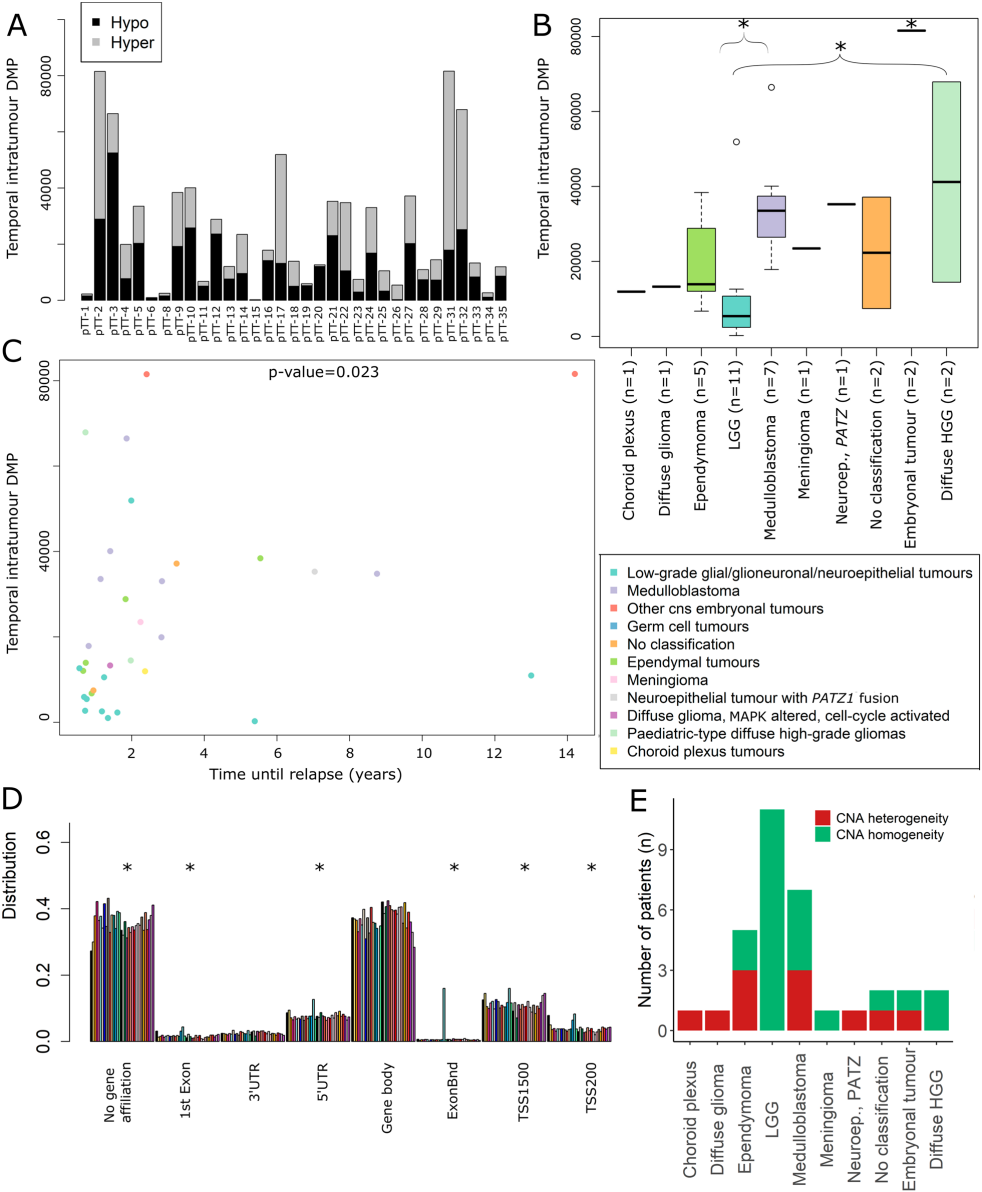
The number of methylation alterations increases over time and with higher-grade tumours. **A** The number of differentially methylated positions (DMPs) between the primary and relapse tumour (Δβ > 0.3) varied between the patients (one stacked bar per patient). Black colour shows the number of DMPs that are hypomethylated in the relapse tumour compared to the primary tumour. The grey colour means number of hypermethylated DMPs in the relapse compared to the primary tumour. **B** The number of DMPs was significantly higher (*p*-value < 0.05, denoted by *) in the methylation superfamily termed other embryonal tumours (AT/RT etc.) than the others except for diffuse HGG. Note that significance could not be calculated against groups with only one sample. Diffuse HGG and medulloblastoma both had significantly more DMPs than LGG. **C** The number of DMPs (y-axis) is significantly increased (*p*-value = 0.023; Cox proportional hazard regression model test) with longer relapse times (x-axis). **D** The DMPs were significantly (*p*-value < 0.01; two-sided wilcox test) enriched in regions that were not associated with a gene and decreased in 1st Exon, 5’UTR, Exon boundaries, TSS1500 (transcription start site and 1500 bp away) and TSS200 (transcription start site and 200 bp away) regions compared to the distribution of the array (first black bar). * denotes significant *p*-value < 0.01. **E** The CNA profiles of the paired primary and relapse samples were inspected visually for differences (gains/amplifications/deletions) and classified as “CNA heterogeneity” or “CNA homogeneity”. The patients are grouped based on the methylation superfamily classification of the primary tumour. Abbreviations: Choroid plexus – Choroid plexus tumours. Diffuse glioma – Diffuse glioma, *MAPK* altered, cell cycle-activated. LGG – low-grade glial/glioneuronal/neuroepithelial tumours. Neuroep. *PATZ* – Neuroepithelial tumour with *PATZ1* fusion. Embryonal tumour – Other embryonal tumours. Diffuse HGG – Paediatric-type diffuse high-grade gliomas

DMP analysis (Δβ > 0.3) of the temporal samples was performed after removal of CpG sites in homozygous deletions (Additional File 6A). The majority of altered CpG sites was hypomethylated in the relapse compared to the primary tumour (19 of 33 patients = 58%; Fig. 6A). The number of DMPs was dependent on the tumour type where high-grade tumours such as atypical teratoid rhabdoid tumours (AT/RT), had significantly more DMPs than all other tumour types except HGG (*p*-value < 0.01; *t*-test). Further, HGG and medulloblastoma respectively had significantly more alterations than LGG (Fig. 6B). The time between the primary and relapse tumour was predictive (*p*-value = 0.02; Cox proportional hazards regression model test) of the number of DMPs, where longer time resulted in more alterations (Fig. 6C). We also verified that differences in tumour cell content did not significantly affect methylation alterations (Additional File 6B). Further, the location of the alterations was similar as in the spatial setting as there were significant reduction in CpG islands, exon and TSS regions, and enrichment in OpenSea and regions without gene affiliation compared to the distribution of CpG sites on the array (Fig. 6D, Additional File 6C).

We noted that the relapse tumour had more CNAs than the primary tumour in around one third of the temporal patients, particularly in ependymomas and medulloblastomas, but not in any of the LGGs in our cohort (Fig. 6E). For instance, the ependymoma pTT-12 had almost no CNAs in the primary tumour whereas the relapse, almost 2 years later, had several deletions (Additional File 7). There was an indication of subclass switch in the relapse tumour, pfa ependymoma subclass 1d (calibrated score 0.52) from subclass 1b (calibrated score 0.98). The tumours with CNA heterogeneity vs CNA homogeneity did not differ in terms of number of DMPs or time to relapse, but our cohort could be too small to detect significant associations.

## Discussion

Paediatric tumours generally have relatively few mutations and genomic alterations, and overall differ substantially compared to the adult counterparts [1, 10]. Epigenetic deregulation, including DNA methylation, is therefore believed to be of particular importance in paediatric cancer. DNA methylation has subsequently proven to be an effective method for tumour classification and further subtyping of tumours into clinically and biologically relevant subgroups [3, 28, 36]. DNA methylation-based classification is already used clinically for certain tumours (e.g. medulloblastoma), but several studies have shown the value of including it upfront in routine clinical use for all paediatric brain tumours [3, 4, 30] (accepted manuscript Schepke et al.). It is therefore essential to verify a homogeneous methylation-based classification spatially within tumours, especially given the recent studies with single-cell approaches that show a higher intratumour heterogeneity in paediatric brain tumours than previously assumed from bulk data analyses [14, 26, 42]. We therefore performed the first study in paediatric brain tumours examining the methylation-based classification in different regions of the same tumour (spatial samples), and over time in paired primary and relapse tumours (temporal samples).

The intratumour methylation superfamily classification of paediatric brain tumours was homogeneous for all included patients in the spatial and temporal study in our cohort that received a successful classification score (≥ 0.9), after exclusion of samples classified as control tissue. Excluding samples purely based on tumour cell content is likely not a wise approach in paediatric brain tumours as certain diagnoses (e.g. some LGG) have relatively low tumour cell content (as low as 45% in this study), but still receive a successful classification score. We therefore made a combined assessment based on the classification, tumour cell content and CNA profile to exclude samples.

Previous intratumour studies on paediatric brain tumours have focused on genomic and transcriptomic heterogeneity rather than epigenetic heterogeneity. Spatial heterogeneity has for instance been reported for paediatric HGG and medulloblastoma regarding CNAs and mutations [12, 24, 41]. Single-cell studies in medulloblastoma have also demonstrated heterogeneity in transcriptomic expression [14, 42]. Paediatric LGG have mainly been studied regarding temporal genomic heterogeneity. A study reported that 11% of LGG differed in *CDKN2A* deletion status in the primary vs the relapse tumour, and that acquisition of the deletion was associated with worse prognosis [21]. We detected no such alterations in our cohort of 11 temporal LGG patients, but we did detect CNA heterogeneity in one of the spatial LGGs. Similar to previous studies [24], we also detected spatial CNA heterogeneity in medulloblastoma.

As mentioned above, intratumour methylation heterogeneity is less studied than genomic heterogeneity. In the relapse setting in medulloblastoma, there has however been reports of methylation subclass switches in rare cases [20], but no details on the methylation alterations. Within adult brain tumours, we previously found spatial intratumour heterogeneity of methylation subclasses in GBM [44] (according to classifier v11b2 and 11b4) and high-grade meningioma, but not in LGG (v12.5) [8]. The subclasses in the paediatric brain tumours examined here were however stable for all spatial and temporal patients (classification score ≥ 0.9; MNP classifier v12.5), which is promising for clinical diagnostics. It should be noted that unfortunately, no paediatric HGG or AT/RT were included in the spatial study due to their rarity and often challenging tumour location. If multiple subclasses were to occur within a single paediatric brain tumour, it would most likely be in high-grade tumours, as we noted that they had the most alterations in the temporal setting.

Our results of homogeneous methylation classification adds to the many studies showing DNA methylation as an effective and useful complement in the diagnosis of paediatric brain tumours [4, 30] (accepted manuscript Schepke et al.). Improved diagnostics leads to a more precise treatment and the time and cost spent on the initial methylation profiling could be balanced out by e.g. reduced follow-up radiology in low-grade tumours. A diagnosis strengthened by methylation classification (and the accompanying CNA profile) may also eliminate the need of costly analyses such as whole-genome sequencing or extensive immunohistochemical workup. The usefulness of the newest version (12.5) of the MNP methylation-based classifier was also highlighted for one of the temporal patients in this cohort (pTT-13), which was diagnosed several years before the release of v12.5. The PAD was a CNS embryonal tumour NOS and the MNP v11b4 classifier weakly assigned the primary tumour as a plexus tumour (calibrated score 0.32) and the relapse tumour as well (calibrated score 0.38). We ran the v12.5 of the classifier for this study and received a strong classification of supratentorial ependymoma *ZFTA* fusion-positive (calibrated score 0.99). If v12.5 of the classifier had been available at the time of diagnosis, it would likely have resulted in a change of diagnosis in favour of ependymoma. This would also have led to a change in treatment, highlighting the value of the new v12.5 classifier.

The threshold for a successful classification in the new v12.5 of the MNP classifier could potentially be lowered from 0.9 to 0.84, which was frequently used for the previous classifier version 11b4 [4]. Considering 0.84 as the threshold for this study would result in very few changes, namely; GU-pBT-88 would have heterogeneous subclasses, and three more samples would be successfully subclassified (GU-pBT-138_1, pTT-35_P and pTT-26_R). Scores below the threshold may also be useful and there were indications of subclass heterogeneity with lower calibrated scores (< 0.9) in a few cases in our cohort both in the spatial and temporal setting. It is as of yet unclear if this represents an actual subclass heterogeneity, or merely that these subclasses need to be further delineated, especially considering that some of them are novel for the v12.5 of the classifier. Most of these subclasses are currently of no importance in clinical treatment of the patients, but may be in the future, in which case the potential heterogeneity/unclear subclassification need to be addressed. We also examined the temporal patients where the PAD was not an exact match in the relapse tumour compared to the primary tumour and found that the methylation classification was more stable and e.g. successfully classified two tumours (pTT-9 as supratentorial EPN, *ZFTA:RELA* fusion-positive, and pTT-21 as a neuroepithelial tumour with *PATZ1* fusion) where the PAD could not provide a specific entity (malignant glioneural and high-grade tumour respectively). The methylation pattern is stable over time, and is a valuable diagnostic tool in the relapse situation as well.

While the overall subclass-specific methylation pattern was intact, we did note site-specific methylation alterations (DMPs) between different regions of the same tumour. The mean number was 7600 in LGG and we observed a trend of more DMPs in medulloblastomas, 17,100 DMPs. It should be noted that the LGG had on average one more biopsy per patient for this analysis, which increases their number of DMPs meaning that differences between the tumour types probably is even larger than the numbers suggest. We have previously reported an average of 21,000 DMPs in adult GBM (grade 4 tumour), 24,000 in adult high-grade meningioma, 17,000 in adult LGG and only 100 in adult low-grade (grade 1) meningioma [8, 44]. The pattern of more alterations with increasing tumour grade was also observed here for the paired primary and relapse paediatric patients, where the aggressive and fast-growing AT/RT and HGG had significantly more DMPs than the LGGs etc. We also noted significantly more DMPs with longer time between the primary and relapse tumour, indicating that the tumour accumulates more alterations with more time. Difference in tumour cell content (both in the spatial and temporal setting) however, did not increase DMPs. The majority of the temporal patients had predominantly demethylated (hypomethylated) CpG sites in the relapse compared to the primary tumour. DNA demethylation has previously been associated with chromosomal instability [35], malignant progression of adult LGG [6, 25], and worse prognosis for adult GBM patients with demethylated promoters in the relapse tumour [19]. A similar scenario could potentially be true for paediatric tumours as well, but would require longer follow-up time and a larger cohort to study.

Methylation alterations occurred in all regions, including CpG islands, exons and TSS, but were significantly depleted in these regions, and significantly enriched in OpenSea and regions not associated with a gene, indicating that alterations are more likely to occur in non-regulatory regions. Almost no DMPs were shared between patients, suggesting that the alterations occur at random sites, but most commonly in non-regulatory regions. Since the superfamily and subclass classification remained homogeneous (based on samples successfully classified with a calibrated score > 0.9), the observed alterations did not affect the CpG sites involved in defining the methylation subclasses, or did not affect enough of them or with large enough methylation differences to induce a switch in subclass. The methylation pattern defining the subclasses were therefore mainly intact across both time and space.

## Conclusions

The methylation-based superfamily and subclass were homogeneous in all spatial and temporal patients in our cohort that were successfully classified (calibrated score ≥ 0.9), demonstrating that methylation-based classification is robust across the tumour and not dependent on the sampled location. We did note indications of heterogeneous methylation subclasses (calibrated scores < 0.9) spatially and temporally, and more studies are needed to determine if that represents true heterogeneity. Further, site-specific DNA methylation alterations occurred, both spatially and temporally, and these alterations were more frequent in high-grade tumours and accumulated over time. Very few DMPs were shared between the patients, and they were enriched in OpenSea, and regions without gene affiliation, suggesting that the alterations are randomly located, but preferentially in non-regulatory regions. The detected site-specific alterations did not affect the methylation-based classification, and the subclass-specific methylation pattern in paediatric brain tumours was thus largely stable across both space and time.

## Supplementary Information

**Additional file 1.** Hierarchical clustering based on the single nucleotide polymorphism sites included on the EPIC methylation array verifies the patient identity for all samples (coloured by their patient identity).

**Additional file 2.** A) Boxplot visualising the classification score for each methylation superfamily. The classification score ranges between 0 and 1, where ≥0.9 is considered a successful classification (indicated by dashed line). B) The superfamily classification score (y-axis) for all samples (spatial and temporal) versus the tumour cell content (x-axis). The samples are coloured according to their methylation superfamily. C) Hierarchical clustering of the top 10000 most variable CpG sites and D) multi-dimensional scaling (MDS) plot of the top 20000 most variable CpG sites of all samples mainly cluster samples according to their methylation superfamily. The legend in D applies to all subfigures. Abbreviations: Choroid plexus – Choroid plexus tumours. Diffuse glioma – Diffuse glioma, MAPK altered, cell cycle-activated. LGG – low-grade glial/glioneuronal/neuroepithelial tumours. Neuroep. PATZ – Neuroepithelial tumour with PATZ1 fusion. Embryonal tumour – Other embryonal tumours. Diffuse HGG – Paediatric-type diffuse high-grade gliomas.

**Additional file 3. ** Sankey plot of the pathological-anatomical diagnosis (left) and the top methylation subclass (right) for included A) spatial samples, and B) temporal samples. Only the primary tumour is included in the B-panel. Note that the A-panel is organised according to patient-ID. NOS – not otherwise specified.

**Additional file 4.** A) Hierarchical clustering of all included spatial samples based on the top 10000 most variable CpG sites. Samples are coloured based on their patient identity (legend for the patient samples in the F-panel applies here as well). B) Phylogenetic tree of GU-pBT-103 and C) GU-pBT-110 based on distance calculations of the top 5000 most variable CpG sites. A sample of paediatric brain tissue is included in the phylogenetic tree as a normal tissue reference. The colour indicates the methylation subclass and the number in parenthesis the classification score. GU-pBT-103 and GU-pBT-110 has indications of multiple subclasses, but not confirmed as the calibrated scores are below 0.9. D) The number of differentially methylated positions (DMPs) within each tumour with and without CNA filter show little differences and the filter is therefore used for DMP analysis. E) Very few DMPs are shared between the patients, but alterations occur F) predominantly in OpenSea regions. * denotes significant (p-value<0.01; two-sided wilcox test) alteration compared to the distribution on the array.

**Additional file 5. ** A) Copy-number alteration (CNA) profiles for the low-grade glioma GU-pBT-93 from two different locations of the tumour (top and bottom), which differ from each other mainly regarding gain of chromosome 12. B) CNA profile from two different locations of the medulloblastoma GU-pBT-109. The biopsies differ regarding gain of chromosome 1q, gain of 15q and focal deletion of 8p.

**Additional file 6. ** A) The number of differentially methylated positions (DMPs) between the primary and relapse tumour (Δβ-value larger than 30% is considered a DMP) for each patient with and without correction for homozygous deletions. B) The number of temporal DMPs (y-axis) is not correlated to the difference in tumour cell content (r=0.24, p-value=0.18) between the primary and relapse sample (x-axis). The patients are coloured according to the best-predicted methylation superfamily. C) Distribution of the temporal DMPs for each patient over the different methylation regions. The black bar to the left in each group shows the distribution of all CpG sites on the EPIC methylation array. * denotes significant (p-value<0.01; two-sided wilcox test) alteration of the temporal DMP in the tumours compared to the distribution of CpG sites on the array.

**Additional file 7. ** Several patients had more copy-number alterations (CNAs) in the relapse tumour compared to the primary tumour. The figure shows an example of an ependymoma where the primary tumour (top) has very few alterations while the relapse tumour (bottom) has acquired more alterations.

## Data Availability

The datasets generated during the current study are available from the corresponding author on reasonable request. Paired medulloblastoma and metastasis samples was also used and is available at the Gene Expression Omnibus; GSE63669 [43].

## Abbreviations

AT/RT: Atypical teratoid rhabdoid tumour
CNA: Copy-number alteration
CpG: Cytosine-phosphate-guanine
DMP: Differentially methylated position
FFPE: Formalin-fixed paraffin-embedded
GBM: Glioblastoma
HGG: High-grade glioma
LGG: Low-grade glioma
MGMT: O6-methylguanine–DNA methyltransferase
MNP: Molecular Neuropathology
PAD: Pathological-anatomical diagnosis
PXA: Pleomorphic xanthoastrocytoma
SHH: Sonic hedgehog
TSS: Transcription start site
WNT: Wingless
