# Supplementary figures and images for: DNA methylation alterations across time and space in paediatric brain tumours
Source: Acta Neuropathol Commun. 2022 Jul 16;10:105. doi: 10.1186/s40478-022-01406-8 (PMC9287974; doi:10.1186/s40478-022-01406-8)

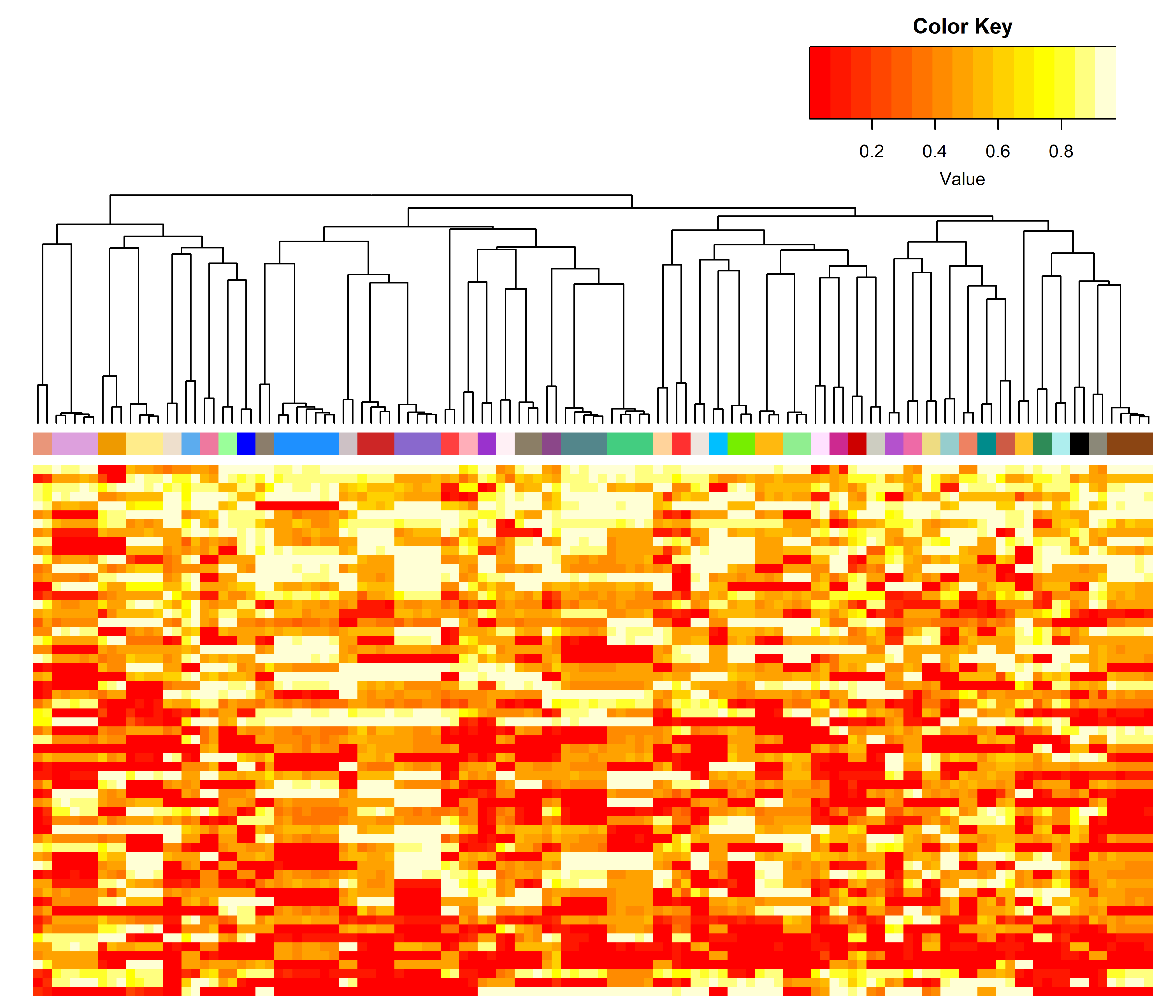

Supplement: Supplementary file 1 — Additional file 1. Hierarchical clustering based on the single nucleotide polymorphism sites included on the EPIC methylation array verifies the patient identity for all samples (coloured by their patient identity). [file 40478_2022_1406_MOESM1_ESM.tiff]

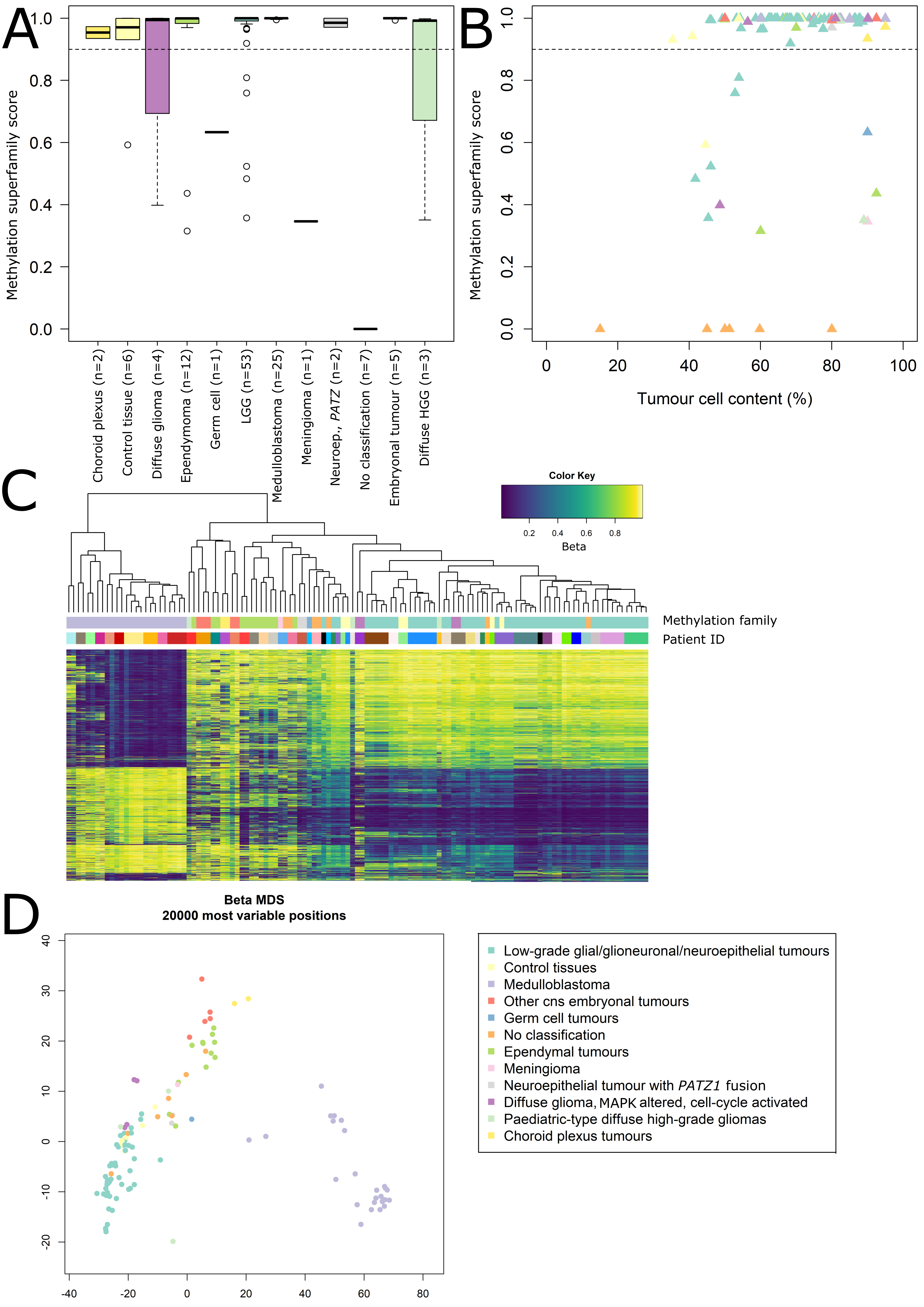

Supplement: Supplementary file 2 — Additional file 2. A) Boxplot visualising the classification score for each methylation superfamily. The classification score ranges between 0 and 1, where ≥0.9 is considered a successful classification (indicated by dashed line). B) The superfamily classification score (y-axis) for all samples (spatial and temporal) versus the tumour cell content (x-axis). The samples are coloured according to their methylation superfamily. C) Hierarchical clustering of the top 10000 most variable CpG sites and D) multi-dimensional scaling (MDS) plot of the top 20000 most variable CpG sites of all samples mainly cluster samples according to their methylation superfamily. The legend in D applies to all subfigures. Abbreviations: Choroid plexus – Choroid plexus tumours. Diffuse glioma – Diffuse glioma, MAPK altered, cell cycle-activated. LGG – low-grade glial/glioneuronal/neuroepithelial tumours. Neuroep. PATZ – Neuroepithelial tumour with PATZ1 fusion. Embryonal tumour – Other embryonal tumours. Diffuse HGG – Paediatric-type diffuse high-grade gliomas. [file 40478_2022_1406_MOESM2_ESM.tiff]

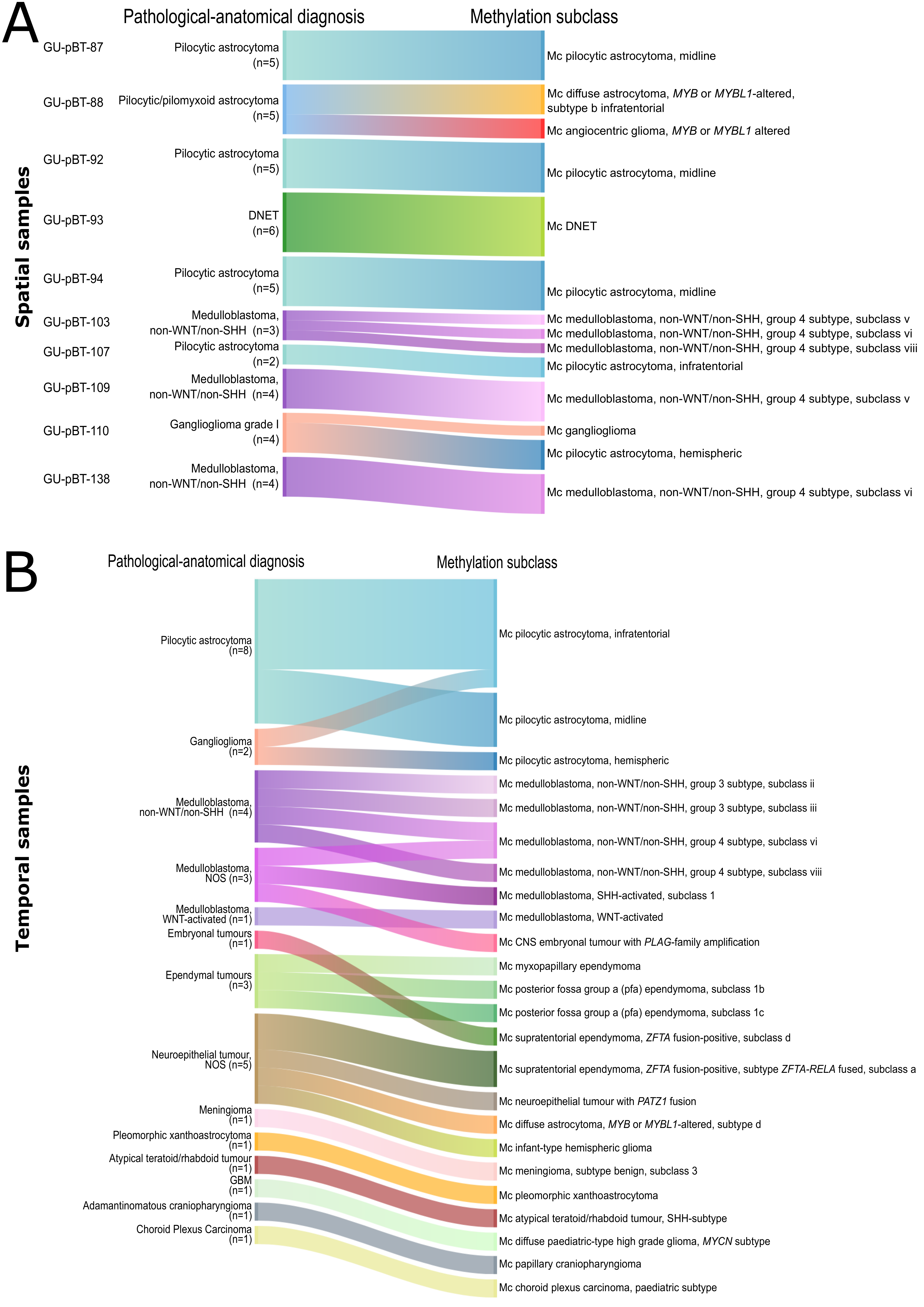

Supplement: Supplementary file 3 — Additional file 3. Sankey plot of the pathological-anatomical diagnosis (left) and the top methylation subclass (right) for included A) spatial samples, and B) temporal samples. Only the primary tumour is included in the B-panel. Note that the A-panel is organised according to patient-ID. NOS – not otherwise specified. [file 40478_2022_1406_MOESM3_ESM.tiff]

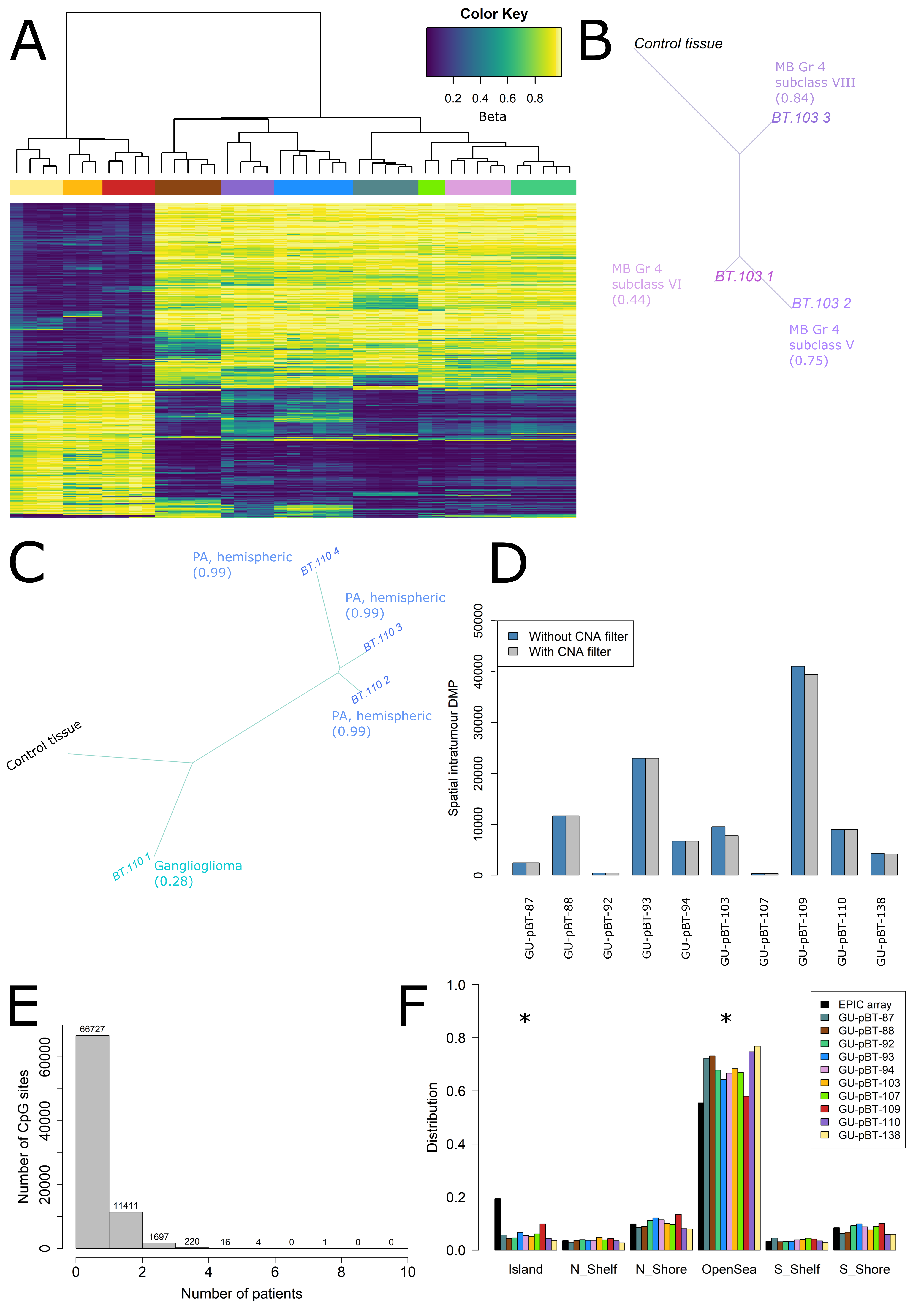

Supplement: Supplementary file 4 — Additional file 4. A) Hierarchical clustering of all included spatial samples based on the top 10000 most variable CpG sites. Samples are coloured based on their patient identity (legend for the patient samples in the F-panel applies here as well). B) Phylogenetic tree of GU-pBT-103 and C) GU-pBT-110 based on distance calculations of the top 5000 most variable CpG sites. A sample of paediatric brain tissue is included in the phylogenetic tree as a normal tissue reference. The colour indicates the methylation subclass and the number in parenthesis the classification score. GU-pBT-103 and GU-pBT-110 has indications of multiple subclasses, but not confirmed as the calibrated scores are below 0.9. D) The number of differentially methylated positions (DMPs) within each tumour with and without CNA filter show little differences and the filter is therefore used for DMP analysis. E) Very few DMPs are shared between the patients, but alterations occur F) predominantly in OpenSea regions. * denotes significant (p-value<0.01; two-sided wilcox test) alteration compared to the distribution on the array. [file 40478_2022_1406_MOESM4_ESM.tiff]

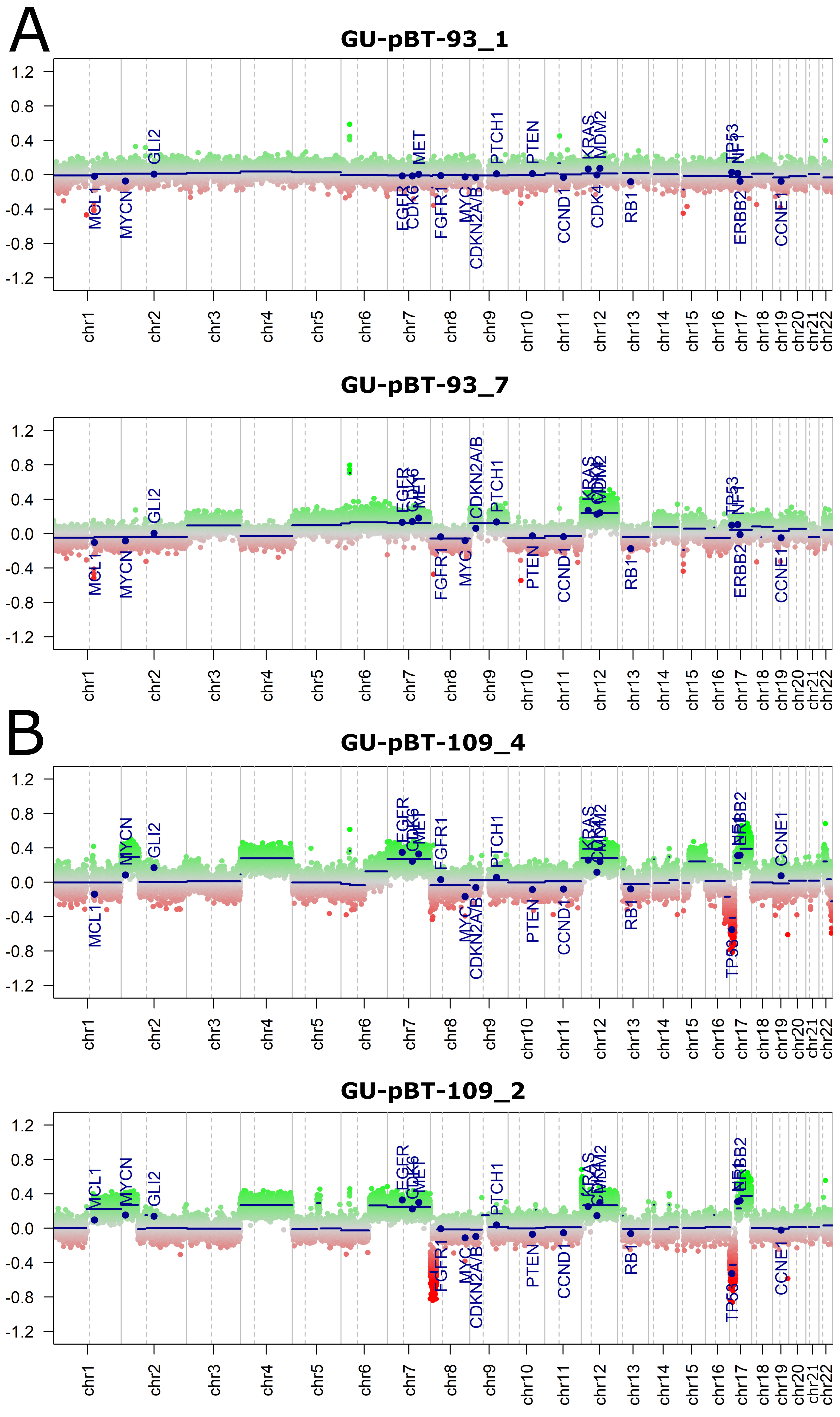

Supplement: Supplementary file 5 — Additional file 5. A) Copy-number alteration (CNA) profiles for the low-grade glioma GU-pBT-93 from two different locations of the tumour (top and bottom), which differ from each other mainly regarding gain of chromosome 12. B) CNA profile from two different locations of the medulloblastoma GU-pBT-109. The biopsies differ regarding gain of chromosome 1q, gain of 15q and focal deletion of 8p. [file 40478_2022_1406_MOESM5_ESM.tiff]

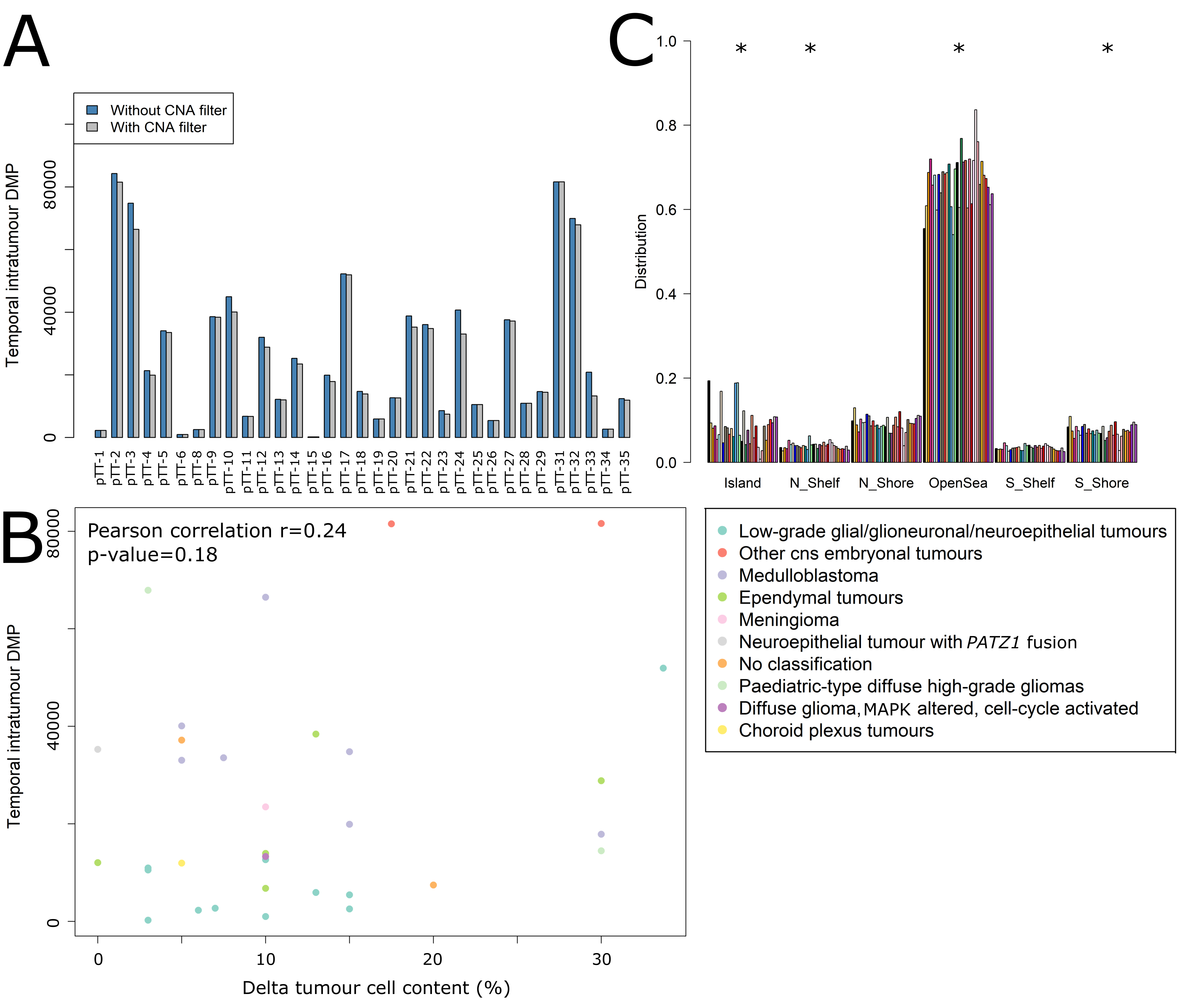

Supplement: Supplementary file 6 — Additional file 6. A) The number of differentially methylated positions (DMPs) between the primary and relapse tumour (Δβ-value larger than 30% is considered a DMP) for each patient with and without correction for homozygous deletions. B) The number of temporal DMPs (y-axis) is not correlated to the difference in tumour cell content (r=0.24, p-value=0.18) between the primary and relapse sample (x-axis). The patients are coloured according to the best-predicted methylation superfamily. C) Distribution of the temporal DMPs for each patient over the different methylation regions. The black bar to the left in each group shows the distribution of all CpG sites on the EPIC methylation array. * denotes significant (p-value<0.01; two-sided wilcox test) alteration of the temporal DMP in the tumours compared to the distribution of CpG sites on the array. [file 40478_2022_1406_MOESM6_ESM.tiff]

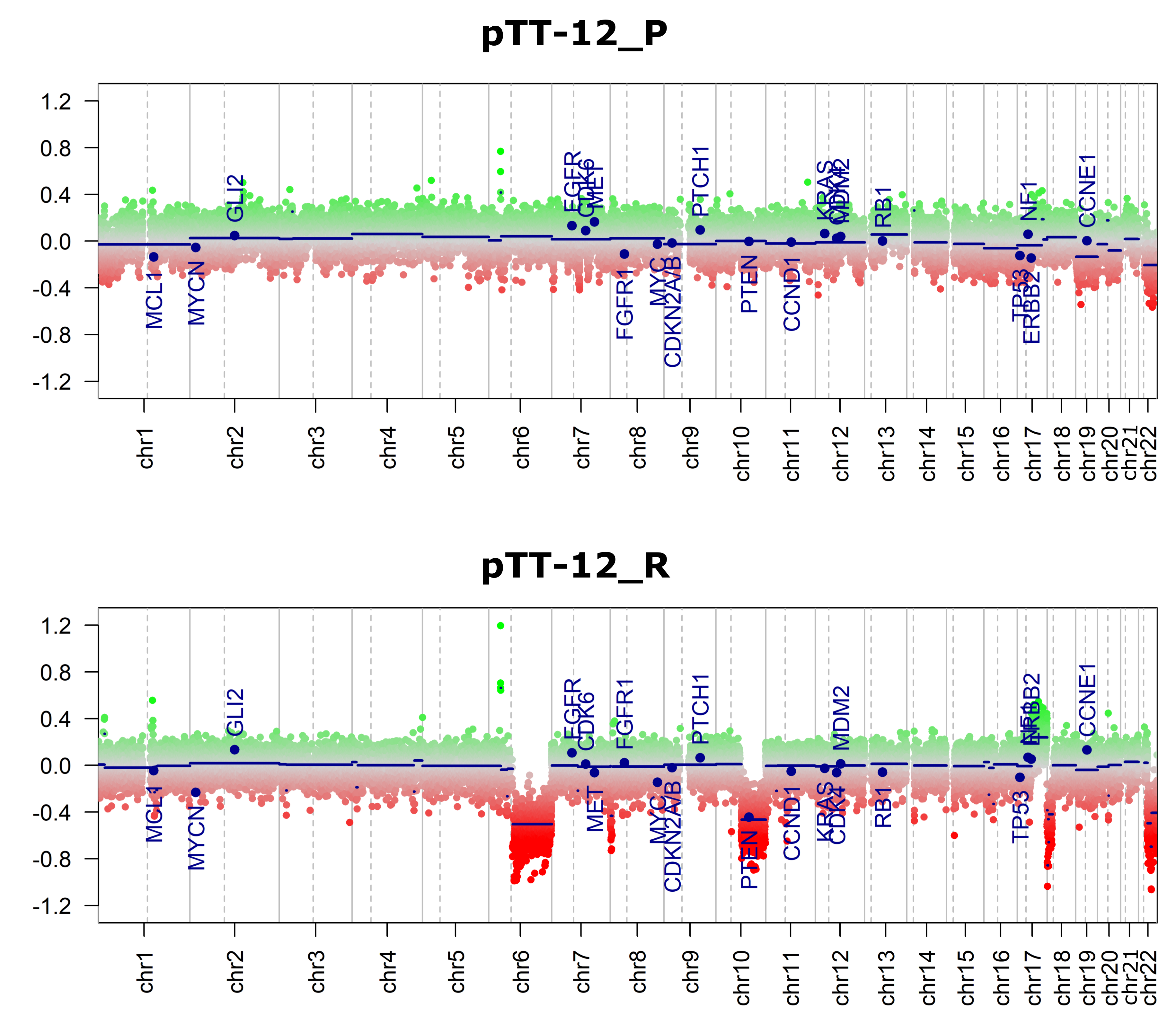

Supplement: Supplementary file 7 — Additional file 7. Several patients had more copy-number alterations (CNAs) in the relapse tumour compared to the primary tumour. The figure shows an example of an ependymoma where the primary tumour (top) has very few alterations while the relapse tumour (bottom) has acquired more alterations. [file 40478_2022_1406_MOESM7_ESM.tiff]
